# Development of the Chinese Family Support Scale in a Sample of Chinese Patients with Hypertension

**DOI:** 10.1371/journal.pone.0085682

**Published:** 2013-12-20

**Authors:** Gang Li, Huanhuan Hu, Zhong Dong, Takashi Arao

**Affiliations:** 1 Institution of Chronic Disease Control and Prevention, Beijing Center for Diseases Control and Prevention, Beijing, China; 2 Laboratory of Exercise Epidemiology, Graduate School of Sport Sciences, Waseda University, Tokorozawa, Japan; 3 Laboratory of Exercise Epidemiology, Faculty of Sport Sciences, Waseda University, Tokorozawa, Japan; University of South Australia, Australia

## Abstract

**Background:**

Despite strong recommendations to involve family social support in hypertension control, few questionnaires have been designed to measure family support in Chinese patients. The Chinese Family Support Scale is a self-rated questionnaire that assesses family support over a 6-month period.

**Methods:**

A total of 282 patients with hypertension participated in this study and 136 of them completed the questionnaire twice within an interval of two to three weeks. Exploratory factor analysis was conducted to assess the structural validity of the scale. Concurrent validity was determined by measuring the correlation between the Chinese Family Support Scale, and Hospital Anxiety and Depression Scale using the Sperman’s Correlation Coefficient. Cronbach’s alpha and intraclass correlation coefficients were employed to evaluate the internal and test-retest reliability of the scale.

**Results:**

Exploratory factor analysis revealed a three-factor solution accounting for 62% of the total variance. The three underlying sub-scale dimensions were kinship, nuclear family, and social resources. Significant correlation (r=-0.266; *p*<0.01) was found between the depression subscales of the Hospital Anxiety and Depression Scale and the extent of support perceived by the patients as measured by the Chinese Family Support Scale. The Chinese Family Support Scale had an acceptable internal consistency (Cronbach’s alpha = 0.84) and test-retest reliability (intraclass correlation coefficient = 0.82).

**Conclusion:**

The study provides preliminary evidence that the12-item Chinese Family Support Scale is acceptable, valid and reliable for measuring the perceived family support in hypertension patients. It is a promising tool which can be easily incorporated into epidemiological surveys.

## Introduction

Hypertension is usually a life-long condition, requiring continuous treatment. Management of hypertension involves substantial daily effort, including antihypertensive medication, blood pressure monitoring, and modification of physical activity, diet, and other daily habits [1]. Such lifestyle changes and coping with the hypertension management, may put the patients at risk of developing mental disorders [2,3]. Given the complexity of hypertension management and possible coexistence of mental disorders, many hypertensive patients may need support to manage their blood pressure successfully. Such support from family, friends, and professional organizations has received great attention in chronic disease care in the last decade [4,5]. A growing body of literature indicates that patients with higher levels of family support would be more likely to exhibit self-care behaviors frequently [6,7]. However, most of these studies focused on diabetes, and limited evidence from studies on patients with hypertension showed that family support might improve therapy compliance and health dietary habits [8,9]. 

In addition to the relationship between family support and self-care, studies demonstrated a link between low levels of family social support and poor mental health [10-12]. Psychological problems like depression and anxiety have been found to be common among hypertensive patients [2,3]. A cohort study showed that increasing levels of anger, decreasing levels of social support, and high anxiety increase the likelihood of women’s development of hypertension in midlife [13]. Considering the effects of family support in improving self-care behaviors and mental health, there has been an urge from researchers to involve family social support in the control of chronic diseases such as hypertension [14,15].

It is estimated that cardiovascular diseases affect 230 million Chinese, out of which 200 million have hypertension [16]. In China, data on the association between hypertension self-care and family support are scarce. A recent systematic review suggested that few studies investigated family support among hypertensive patients, and the quality of such studies, was generally poor [17]. Lack of appropriate scales for measuring family support may be one of the reasons contributing to this. In the past decades, several family support scales have been developed, most of which were developed in the western countries [18,19]. In China, families are tied closely by blood relationship and the “family first” ideology may motivate family members to help relatives suffering from a disease [20]. This traditional culture is different from that seen in the western countries, which makes it difficult to use these scales with the Chinese population. To know the association between family support, self-care, and outcome of hypertension, it is essential to have a reliable and valid family support scale that can be used with Chinese patients.

Family support represents complex social ties that are difficult to define and measure objectively. The exact elements that compose family support, and their relative importance, may vary across individuals and medical conditions. Some of the existing questionnaires limited family support to family members related by blood, while others used the term “family support” to include the support provided by the immediate family, extended family and other relatives, as well as friends [18,19]. In this study, we paid particular attention to the role of family members, relatives, and friends. In addition, we hypothesized that family social support affects each self-care behavior differently. For instance, support from family members may be more important for self-care behaviors within the daily routine, like meal planning [9]. On the other hand, performance of some self-care behaviors, such as medication adherence or blood pressure monitoring, may depend on factors external to the family members, such as professional agencies [21,22]. This scale was designed to investigate the relationship between family social support and self-care behaviors, therefore, some social support resources such as professional agencies and social organizations were included, as they may be important to gain a complete understanding of the performance of self-care behaviors. 

To the best of our knowledge, until this study was conducted, there was no validated family support scale for Chinese hypertensive patients for assessing the sense of support perceived from different family members and non family members by Chinese hypertensive patients. The Chinese Family Support Scale (CFSS) was developed in the present study to provide an instrument that is easy to use and interpret in epidemiological surveys with patients. Further, the objective of this study was to examine the reliability and validity of the CFSS. 

## Methods

The Ethical Review Board in Waseda University granted permission to conduct this study. Written informed consent was obtained from all the participants prior to data collection. Participants were informed that they could stop the interview at any time and decline to answer questions without having to give any reasons for the same.

### The Chinese Family Support Scale (CFSS)

The CFSS developed in this study is a 12-item measure of how helpful different sources of family support have been to the patients with hypertension (File S1). To avoid transient disturbances and reduce recall bias, the CFSS assesses the support that patients with hypertension perceived during the 6 months prior to data collection.

### Instrument development

Items in the CFSS were derived from two sources: a review of previous family support scales reported in the literature [18-20] and discussions with public health professionals. At first, family support resources were classified into four broad categories: family members, relatives, friends, and social organizations, and the items that fell into these categories were listed. Thus, a 17-item pool was built based on the literature review and existing knowledge about family support. These items were evaluated and discussed with the authors and two other public health professionals, during which each item was evaluated for its relevance to the concept of family support (0=not relevant, 1=a little relevant, 2=relevant, 3=very relevant). Following this, an average relevance score was calculated for each item, and items that scored 2 or more were retained in the CFSS. Data saturation was achieved after the second focus group meeting, as there was no recommendation for further inclusion or exclusion of items. Thus, 12 items were selected from the 17-item pool, which appeared in the final tool. The CFSS items and instructions were drafted according to the recommendations regarding cognitive burden, response format and layout, and question order [23,24]. The twelve items assessed the perceived support from five key support resources: family members (4 items), formal kinship (2 items), informal kinship (3 items), social organizations (2 items), and professional agencies (1 item). 

### Scoring

The CFSS consisted 12 items rated on a 6-point Likert scale, ranging from “Not available” (0) to “Extremely helpful” (5). Participants had to circle the relevant response for each item. These scores were summed to yield a total CFSS score, which ranged from 0–60, a higher score indicating better family support.

### Participants

The questionnaire survey was undertaken in a local community health clinic in Beijing, China, in 2012. Eligible participants were aged 35 years and above, and had been diagnosed with hypertension at least 12 months before data collection. Participants who could not communicate effectively with the study personnel or provide informed consent were excluded. Costello and Osborne [25] empirically tested the effect of the sample size on the results of factor analysis. They reported that 70% of the samples with a ratio of 20:1 (sample size: number of items) resulted in a correct factorial structure. This corresponded to 240 patients for a 12-item questionnaire. We recruited subjects for this study through a community health clinic which is a public medical center providing medical and public health services to civilians. A total of 890 hypertension patients were registered in the health clinic. Physicians screened the registered patients for eligibility for the study and 143 patients without contact information were excluded. Out of the remaining 747 patients, 61.0% (456/747) of them met the inclusion criteria, were invited to participate in this study, and 40.0% (299/747) accepted the invitation.

All interviews were conducted by trained interviewers at the study site by following an interview guide. Interviewers were trained on all study protocol and interview techniques before starting field work. Each interview lasted for an average of 10 minutes.

### Assessment of validity and reliability of the CFSS

A cross-sectional design was used to assess the reliability and validity of the CFSS in a hypertensive population.

### Assessment of validity

To assess the concurrent validity of the CFSS, the Hospital Anxiety and Depression Scale (HADS) [26-28] was used as a criterion measure. Concurrent validity was examined by using the Spearman’s correlation coefficient between the CFSS and HADS. To date, no tool has been identified as the most appropriate for measuring family support among patients with a chronic disease. It has been suggested that there is an important correlation between the support by family, peer and social organizations, and psychological well-being [10-12]. The HADS is widely used as a screening measure for both, dimensional and categorical aspects of anxiety and depression. 

Construct validity was examined by factor analysis of the internal structure of the test. Prior to performing factor analysis, the suitability of the data for such analysis was assessed using the Kaiser-Meyer-Olkin (KMO>0.6) method and Bartlett’s test of sphericity (*p*<0.05) [29]. Exploratory factor analysis was performed on the items to test the CFSS underlying dimensions of the CFSS. A principal component analysis with varimax rotation was performed to extract the factors, and factors with an eigenvalue ≥1.0 were kept as part of the factor structure. This scale was hypothesized to reflect a three-factor model of family support, assessing the following subscales: kinship (items: 1, 2, 3, 4), nuclear family (items: 5, 6), and social resources (items: 7, 8, 9, 10, 11, 12).

### Assessment of reliability

To examine the test-retest reliability of the CFSS, data were re-collected after a two or three week interval from half the patients who were selected from those who had finished the first questionnaire, using convenience sampling. At the end of the first interview, 207 patients were asked if their blood pressure had remained stable for the previous month and if they would be willing to participate in a retest review. When the retest interview quota was complete, the reaming 75 patients were not asked to participate in a retest review. Test-retest reliability was assessed with intra-class correlation coefficient (ICC), where an ICC value of 0.40 represented moderate, 0.60 reflected good, and 0.80 reflected high agreement between the two test situations [30].

The reliability of the scale was examined using Cronbach’s alpha for internal consistency and the Guttmann’s “split-half” reliability. Internal consistency is considered acceptable if the Cronbach’s alpha coefficient is greater than 0.70 [31]. 

### Other measurements

In addition to above-mentioned family support, anxiety and depression measures, demographic information was also collected in the questionnaire including the respondent’s age, sex, education level, occupation and marital status (married, widowed, divorced/separated and unmarried) as well as duration since hypertension was diagnosed.

### Data management and statistical analysis

Data were double-entered and crosschecked using the statistical software Epi Info version 6. Descriptive statistics such as means, standard deviations, medians, percentages and range were used where appropriate. Values were considered statistically significant at *p*<0.05. All statistical analyses were performed using IBM SPSS, version 19 (SPSS Inc., Chicago, IL, U.S.A.).

## Results

### Sample characteristics

Among the 465 eligible hypertension patients, 64.3% (299/645) participated in the survey. Among these, 60.6% (282/465) of them completed the questionnaire and 17 of them provided incomplete answers. Of the 144 patients invited to complete a second questionnaire to assess the test-retest reliability, 94.4% (136/144) of them provided complete answers.

Table 1 displays characteristics of the study sample. Of the 282 respondents, 72.3% were female, and 70.6% reported to have received below 6 years of education. Mean age was 62.8±7.9 years (range: 35–83 years). Participants reported years of hypertension in the range of 1–41 years, with a mean of 7.9 ±6.7years. The mean HADS score was 8.15±6.38. The full-scale Cronbach’s alpha for the HADS was 0.890, was 0.712 for the HADS depression subscale, and 0.773 for the HADS anxiety subscale in our sample. There were no statistically significant differences in age, level of education, anxiety and depression, and duration of hypertension between the test and retest group. 

**Table 1:** Characteristics of the sample.

	n (%) N=282
**Age**	
35-64	158 (56.0)
65-83	124 (44.0)
Mean (SD)	62.8 (±7.9)
**Gender**	
Male	78 (27.7)
Female	204 (72.3)
**Level of education**	
≤6 years	199 (70.6)
>6 years	83 (29.4)
**Marital status**	
Married	250 (88.7)
Others	32 (11.3)
**Annual family income**	
<50,000 yuan	274 (97.2)
≥50,000 yuan	8 (2.8)
**Years of hypertension, Mean (SD)**	8.2 (±7.1)
**HADS, Mean (SD)**	8.15 (±6.38)
HADS depression, Mean (SD)	4.02 (±3.48)
HADS anxiety, Mean (SD)	4.11 (±3.73)

### Validity

#### Concurrent validity

The CFSS was found to have significant correlation with the HADS (Table 2). There were significant correlations between the CFSS and the full-scale HADS scores (r=-0.169; *p*<0.01), and the HADS depression subscale scores (r=-0.266; *p*<0.01). The negative correlation coefficients indicated that higher levels of depression were related to poorer support. No statistically significant correlations were found with the HADS anxiety subscale scores.

**Table 2:** Spearman correlations of the association between the CFSS and the HADS.

CFSS	HADS		
	Anxiety subscale	Depression subscale	Total scores
Kinship	-0.081	-0.141^*^	-0.119^*^
Nuclear family	-0.039	-0.212^**^	-0.133^*^
Social resources	-0.039	-0.246^**^	-0.151^*^
Total scores	-0.049	-0.266^**^	-0.169^**^

Note. ^*^ *p*<0.05; ^**^ *p*<0.01

#### Construct validity

Both the KMO value (0.85) and the statistical significance of the Bartlett’s test of sphericity (χ^2^=1422.34; *p*<0.001) supported that the data were appropriate for exploratory factor analysis. The result of the factor analysis for the CFSS has been presented in Table 3. Our factor analysis revealed a three-factor solution that accounted for 62% of the variance as follows: Factor 1, 41.1%; Factor 2, 10.1%; and Factor 3, 11.2%. The CFSS items 7 and 8 were observed to load on factor 1 and factor 3; item 9 was observed to load on factor 2 and factor 3. These factors will henceforth be referred to as subscales.

**Table 3:** Factor loading of the CFSS items after varimax rotation.

Items	Factor 1	Factor 2	Factor 3
1 Your parents	0.835	0.025	0.050
2 Your spouse or partner’s parents	0.847	0.038	0.029
3 Your relatives	0.534	0.385	0.323
4 Your spouse or partner’s relatives	0.606	0.454	0.315
5 Your spouse or partner	0.157	0.739	-0.059
6 Your children	0.011	0.766	0.122
7 Your friends	0.496	0.398	0.562
8 Your spouse or partner’s friends	0.508	0.430	0.538
9 Co workers	0.346	0.505	0.470
10 Community organizations	0.264	0.105	0.727
11 Professional agencies	-0.184	0.275	0.614
12 Other social organizations	0.111	-0.204	0.708

### Reliability

#### Test-retest reliability

Retests for reliability were completed by 136 patients who completed the first questionnaires. The ICC was 0.820 for the CFSS total scores, 0.789 for the CFSS-kinship, 0.662 for the CFSS-nuclear family, and 0.864 for the CFSS-social resources. The ICC of individual item ranged from 0.628 to 0.862. All of these ICC scores indicate good to excellent reliability range.

#### Internal consistency reliability

The internal consistency of the CFSS was assessed with Cronbach’s alpha and was verified after splitting the sample (Guttmann’s “split-half”). Cronbach's alpha for the total score was 0.840 and the total score split-half was 0.750, representing an acceptable internal consistency. The alpha was 0.794 for the CFSS-kinship, 0.552 for the CFSS-nuclear family, and 0.798 for the CFSS-social resources. Except for items 5 and 11, the removal of one item resulted in lower alpha values in the case of all other items (Table 4). Replacing item 5 or 11 was found to increase the scale’s validity, however, without important differences. The item-total correlations coefficients were above 0.20, which is recommended as the minimum value for including an item in a scale. The results indicated that the scale does not need any modification. 

**Table 4:** Reliability analysis based on the corrected item-total correlation and Cronbach’s alpha coefficient if item deleted.

Items	Corrected Item-Total Correlation	Cronbach’s Alpha if Item Deleted
1 Your parents	0.449	0.832
2 Your spouse or partner’s parents	0.463	0.832
3 Your relatives	0.605	0.821
4 Your spouse or partner’s relatives	0.710	0.815
5 Spouse or partner	0.377	0.847
6 Your children	0.408	0.839
7 Your friends	0.753	0.810
8 Your spouse or partner’s friends	0.769	0.811
9 Co workers	0.660	0.819
10 Community organizations	0.534	0.826
11 Professional agencies	0.308	0.844
12 Other social organizations	0.282	0.843

## Discussion

The CFSS was designed to assess the family support perceived by patients with hypertension, using a number of items to cover relevant aspects of support resources and simple response options. This was the first study to show that the 12-item CFSS demonstrated evidence of reliability and validity in measuring the support hypertension patients perceived.

The results of the factor analysis showed that all items loaded onto three different factors. Parents and relatives loaded together on kinship support (Factor 1), spouse and children also loaded together on nuclear family support (Factor 2), and social agencies, friends, and co-workers/neighbors together loaded on social support (Factor 3). Items referring to friends loaded on both, factor 1 and 3, while the item referring to co-workers/neighbors loaded on both, factor 2 and 3. As these sources of support are often not considered as family members, they may have reflected a source of social support. In the current study, parents were loaded together with relatives, and spouse was loaded together with children. This result may be explained by the characteristics of our sample and the culture-specific nature of the Chinese family system [20]. In our sample, nearly 70% of the participants were aged 60 or above, and among these older patients (≥60 years old), more than three-quarters of their parents were dead. These older patients were more likely to live with their adult children, and receive support from their children and spouse, rather than from their parents who were either dead or too old to provide support. Due to this, our findings were similar to those reported from another study carried out with Chinese patients [20], but the findings from the factor analysis may be sample specific. This suggests that future studies with younger patients may show different results. 

The concurrent validity of the CFSS was examined in relation to the HADS. Findings demonstrated that the CFSS was negatively correlated with the depression subscale of HADS, as established in the literature, while it was not correlated with the anxiety subscale of HADS. The correlation between the CFSS and HADS was not strong (0.169 and 0.266), which may be due to the context in which HADS was used. If a similar family support scale was chosen as a test of concurrent validity of the CFSS, the strength of the correlation may be stronger. Numerous studies have demonstrated an association between family support and depression [32-34]. A 23- year follow up study found that higher family support was associated with less depression and it predicted a steeper trajectory of recovery from depression [35]. Findings reported from various studies that invested the effects of social support on anxiety showed inconsistent and conflicting findings [32,36-39]. The potential reasons for this are unclear. It appears that different types of support (such as instrumental, emotional, and informational) have different effects on individuals [4,5,14,15,40-42]. The current scale assesses only perceived disease-specific support and does not distinguish between the recognized types of support. Future studies that measure these specific types of support may be needed to explain the results reported in the current study and previous studies.

Overall, the reliability of the total and subscale scores was good. For internal consistency, the CFSS total score exceeded the alpha standard of 0.7 for most scales. A lower alpha coefficient for the CFSS-nuclear family was possibly due to the limited items in this construct. It is recommended that a 2 to 4 week interval between measurements is adequate for the test-retest. In this study, we used an interval of 2 to 3 weeks for this reliability. Patients were selected from those who were considered stable before taking the scale for the second time. The CFSS showed good to excellent reliability, indicating that the CFSS scores are stable over time.

This scale has many potential applications for hypertension control. For instance, it can be utilized to identify specific situations in which patients may have problems with family support. As a research tool, it can provide a valuable outcome variable. For instance, family support can be assessed over time in response to mental health, self-care behaviors, and hypertension control. It may also be used in studies that seek to understand mediators or moderators of hypertension control. Finally, as a research tool, it can be used to assess the effectiveness of interventions or programs designed to enhance patients’ family support.

### Limitations of the study

Several limitations also exist in this study. Out of the 890 patients, 31.7% (282/890) completed this survey. The subjects who agreed to participate in this study could be different from those who did not participate. The sample contained more women than men. Further, the generalizability of findings might be limited to the adult population of 35 years and above. The measures were administrated to participants by interviewers. In such situations, people may like to report desirable results, however, given the age and level of literacy of the sample, there was little choice but to collect the data through self-report techniques. 

## Conclusions

The findings from this study aimed to determine the validity and reliability of the CFSC and indicated that it is a reliable and valid measure for research and clinical practices. It is a promising tool that can be easily incorporated into epidemiological surveys.

## Supporting Information

File S1 **Chinese Family Support Scale.**
(DOCX)
