# Supplementary material for: Development of the Chinese Family Support Scale in a Sample of Chinese Patients with Hypertension
Source: PLoS One. 2013 Dec 20;8(12):e85682. doi: 10.1371/journal.pone.0085682 (PMC3869941; doi:10.1371/journal.pone.0085682)
Supplement: File S1 — Chinese Family Support Scale. (DOCX) [file pone.0085682.s001.docx]

## Chinese Family Support Scale

| **Family support scale** | | | | | |
| --- | --- | --- | --- | --- | --- |
| The purpose of this survey is to understand the support you perceived for hypertension control from your family members, friends or other social agencies during the previous 6 months  Please read the items and rate how you feel about each item. | | | | | |
|  | Not available | Not at all helpfull | Sometimes helpful | Generally helpfull | Extremely helpful |
| 1 Your parents |  |  |  |  |  |
| 2 Your spouse or partner’s parents |  |  |  |  |  |
| 3Your relatives |  |  |  |  |  |
| 4Your spouse or partner’s relatives |  |  |  |  |  |
| 5Spouse or partner |  |  |  |  |  |
| 6Your friends |  |  |  |  |  |
| 7Your spouse or partner’s friends |  |  |  |  |  |
| 8 Your children |  |  |  |  |  |
| 9 Co workers |  |  |  |  |  |
| 10Community organizations |  |  |  |  |  |
| 11Professional agencies |  |  |  |  |  |
| 12Other social organizations |  |  |  |  |  |
